# Paraphilic disorders: from the past to the current perspective – a review of the state of the art

**DOI:** 10.1192/j.eurpsy.2025.2302

**Published:** 2025-08-26

**Authors:** M. M. Figueiredo, A. F. Reis, T. Cardoso, V. Vila Nova

**Affiliations:** 1 Centro Hospitalar Setúbal, ULS Arrábida, Setúbal, Portugal

## Abstract

**Introduction:**

Sexuality is a central aspect of human life, encompassing its complexities and diversities. In the field of Psychiatry, Paraphilic Disorders remain a subject of ongoing controversy, including debates over the definition of paraphilia itself.

**Objectives:**

The present work aims to present a review of the current state of the art regarding the evolution of Paraphilic Disorders, analyzing the evidence that supports this diagnosis, and promote the importance of evolution, understanding and non-stigmatization of sexualities.

**Methods:**

Evidence-based review, using a PubMed research and selection of the most relevant studies on this topic, published in the last decade.

**Results:**

An evolution was evident in the classification systems, where initially a pathologization of non-normative sexual practices predominated. Homosexuality and masturbation were understood as diseases, and as such susceptible medical and social control.

In DSM III, the concept of paraphilia, sexual arousal outside normal activity patterns, was introduced in an attempt to reduce stigmatization. But only in DSM V was the distinction made between Paraphilia and Paraphilic Disorder, the latter as causing suffering or dysfunction to the individual or others. This distinction marked a change in paradigm.

**Conclusions:**

The definition and categorization of Paraphilic Disorders in the main classification systems has been influenced by changes in sexual and social norms over time, highlighting their influence on the recognition and treatment of paraphilias.

The view of human sexuality continues to be deeply marked by heteronormativity and reproduction as a social and cultural model, causing sexual practices outside this standard to be labeled as pathological. These diagnoses tend to focus on specific behaviors rather than considering the complexity of each person’s sexual experiences, and to be based on prejudices and stereotypes rather than robust scientific evidence.

**Disclosure of Interest:**

None Declared

